# Tumor Targeted Nanocarriers for Immunotherapy

**DOI:** 10.3390/molecules25071508

**Published:** 2020-03-26

**Authors:** Alejandro Baeza

**Affiliations:** Department of Aerospace Materials and Production, School of Technical Aeronautical and Space Engineering, Technical University of Madrid, 28040 Madrid, Spain; alejandro.baeza@upm.es

**Keywords:** nanomedicine, immunotherapy, cancer therapy, drug delivery

## Abstract

The paramount discovery of passive accumulation of nanoparticles in tumoral tissues triggered the development of a wide number of different nanoparticles capable of transporting therapeutic agents to tumoral tissues in a controlled and selective way. These nanocarriers have been endowed with important capacities such as stimuli-responsive properties, targeting abilities, or the capacity to be monitored by imaging techniques. However, after decades of intense research efforts, only a few nanomedicines have reached the market. The reasons for this disappointing outcome are varied, from the high tumor-type dependence of enhanced permeation and retention (EPR) effect to the poor penetration capacity of nanocarriers within the cancerous tissue, among others. The rapid nanoparticle clearance by immune cells, considered another important barrier, which compromises the efficacy of nanomedicines, would become an important ally in the fight against cancer. In the last years, the fine-tuned ability of immune cells to recognize and engulf nanoparticles have been exploited to deliver immunoregulating agents to specific immune cell populations selectively. In this work, the recent advances carried out in the development of nanocarriers capable of operating with immune and tumoral cells in order to orchestrate an efficient antitumoral response will be presented. The combination of nanoparticles and immunotherapy would deliver powerful weapons to the clinicians that offer safer and more efficient antitumoral treatments for the patients.

## 1. Introduction

The paramount discovery of the passive accumulation of nanoparticles in solid tumors carried out by Maeda and Matsumura a few decades ago [1] opened a new way to treat these malignancies. This phenomena, called enhanced permeation and retention (EPR) effect, is due to the high porosity of the tumoral blood vessels which allows the extravasation of the nanoparticles once they arrive at the diseased tissue in combination with impaired lymphatic drainage within the tumor, that enhances the accumulation of the nanomedicines in the malignancy [2]. Thus, simply by loading the antitumoral drugs inside nanometric carriers, it would be possible to deliver them directly and specifically to the diseased tissues, which would significantly reduce the toxicity associated with the application of these agents. The simplicity and elegance of this finding triggered the development of a wide number of different nanocarriers from simple organic or inorganic nanocarriers as liposomes [3], polymeric [4], mesoporous silica [5], or metallic nanoparticles [6], just to name a few of them, to complex hybrid nanodevices capable to release their payloads in response to different stimuli (pH, redox conditions, enzymes, light, magnetic fields, among others) [7]. Most of these systems have shown excellent antitumoral properties in preclinical assays both in vitro and in vivo, inducing selective tumoral cell elimination and increasing tumoral growth inhibition. However, only around 50 nanomedicines have reached clinical practice [8]. The reasons for this disappointing outcome are varied [9]. Firstly, the EPR effect is common in xenograft mice models but is not ubiquitous in human malignancies. Additionally, it is highly dependent on the tumoral type and even shows significant variations within the same solid tumor and also during the treatment [10]. A recent study has analyzed the results published during the last 10 years about nanoparticle accumulation in solid tumors, concluding that only 0.7% of the administered nanoparticles are delivered to the diseased tissue [11]. This result reflects the need for more research to enhance particle accumulation in the target tissue. Secondly, once the nanoparticle is extravasated in the tumoral tissue, it forms a dense extracellular matrix enriched in collagen, which should overcome. Thus, the diffusion of the nanoparticle is strongly hampered, being mainly located in the tumor periphery, which strongly limits its therapeutic effect [12]. Different strategies to increase the nanomedicines penetration have been proposed, such as the attachment of proteolytic enzymes on the nanocarrier surface [13] or the use of ultrasounds to propel them within the tissue [14]. Despite the promising results yielded by these approaches, their clinical application should be evaluated. Thirdly, solid tumors are not composed of a homogeneous mass of tumoral cells, but they are complex tissues that contain a myriad of different cell populations [15]. Therefore, the nanocarrier should be endowed with the ability to recognize their target. This property is usually achieved by the use of active targeting strategies, which consist of the attachment on the particle surface of small molecules, proteins, or oligonucleotide chains, known as targeting groups, which binds specifically with certain membrane receptors overexpressed by the tumoral cells [16]. Nanocarriers decorated with these targeting moieties have achieved excellent selectivity to deliver therapeutic agents to tumoral cells [17] and to provide information about the cancerous tissue by different imaging techniques [18]. Unfortunately, the use of these targeting groups can compromise the penetration of the nanocarrier within the tissue due to the binding site barrier effect [19]. This effect is caused by the strong retention of the nanoparticles by the first tumoral cell line close to the tumoral blood vessels. This undesired phenomenon would be alleviated using encrypted targeting moieties, which are sequentially activated only in the tumoral tissue [20]. However, these approaches have been evaluated in preclinical settings, and their clinical suitability is still untested. Finally, throughout this complicated journey, nanoparticles should evade their capture by immune cells, which are finely trained to recognize all types of exogenous bodies, as is the case of nanoparticles. Many efforts have been devoted to avoiding immune nanoparticle capture, from the surface decoration with hydrophilic polymers as polyethylene glycol (PEG) [21], to the use of biological membranes of red blood cells [22] or leukocytes as camouflage against the immune system surveillance [23]. Despite these efforts, a significant extent of the injected nanoparticles ends cleared by immune cells, which reduces the amount of them that reach tumoral cells. In recent years, this weakness has begun to be considered, in fact, a powerful strength of nanoparticle applications because their unique capacity to interact with immune cells can be exploited to induce potent immune responses against solid tumors. Thus, the paradigm has been shifted from working against biology, trying to overcome the biological barriers mentioned above, to working with biology, which attempts to exploit the inherent characteristic of nanoparticles in our favor, as it has been excellently reviewed elsewhere [24]. Immune cells have evolved over millennia to recognize viruses and bacteria, which present sizes in the nano- and microscale, respectively. Therefore, they are also well-skilled to interact with nanoparticles, and this fact can be exploited to modulate its function through the careful design of the nanocarriers. One of the most studied approach to trigger self-sustained immune responses against tumoral cells employing nanoparticles is vaccination. In this strategy, the nanocarriers are loaded with tumor antigens and immunostimulating agents to elicit the antitumoral immune response [25]. In these strategies, the nanomedicines are usually targeted to antigen-presenting cells (APC) residents in lymph nodes, releasing their payload within these cells inducing their maturation [26]. One important problem associated with this strategy is the high tumor heterogeneity, which could make the immune response weak if only a few antigens are employed. One alternative consists of the use of nanoparticles, which can enhance the natural response of immune cells against the specific tumoral antigens, which are continuously released in the tumoral tissue in order to trigger an in situ vaccination. Additionally, nanoparticles can be loaded with different drugs to induce several effects at the same time, such as tumoral cell elimination that enhances tumoral antigen release and immunostimulating agents, which induce immune cell maturation. Another approach is to use nanoparticles to remove the immunosuppressive environment, usually present in tumors. These strategies operate in the tumoral tissue, not in the lymphatic nodes. This review will be focused on the application of these tumor-targeted nanoparticles capable of triggering efficient and selective antitumoral responses.

## 2. The Cancer-Immunity Cycle

During cancer progression, tumoral cells undergoing an evolutionary process operated by immune cells known as tumor immunoediting [27]. This process comprises the three E’s phases: Elimination–Equilibrium–Escape [28]. Tumoral cells are mutated versions of healthy ones, which are recognized in early stages by immune cells and, therefore, are efficiently destroyed (Elimination). This preliminary stage corresponds to the elimination phase. At a certain point, tumoral cells achieve the ability to elude immune surveillance, being capable of remaining alive in the host (Equilibrium). This is the longest phase, which can last for years or even decades. At this stage, the immune system prevents tumor outgrowth but maintains residual tumoral cells in a functional dormancy or latent phase. Finally, in the Escape phase, tumoral cells acquire the ability to circumvent the immune system being able to escape to its control. This process can happen due to diverse reasons such as cell alterations that reduce the amount of tumor antigens on the cell membrane, compromising the recognition process by immune cells, acquired higher resistance to the cytotoxic effect of the own immune cells or development of the immunosuppressive environment in the tissue, among others [28].

**Figure 1:**
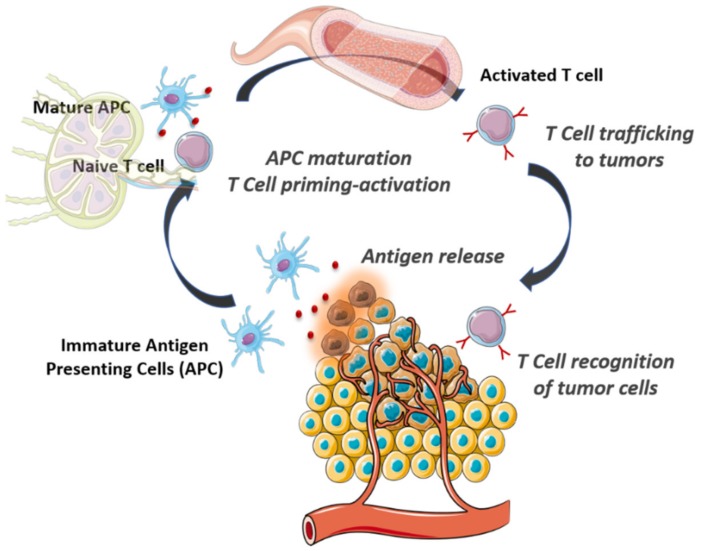
Cancer-immunity cycle.

Immunity against cancer requires the iterative evolution of a series of stepwise steps known as the cancer–immunity cycle (Figure 1) [29]. Tumoral antigens released by death tumoral cells are captured by immature antigen-presenting cells (APC), which are specialized cells of innate immune system cells. If this process is accompanied by the presence of immunostimulating cytokines and factors, APC experience maturation being able to present the captured antigens on their surface. These cells travel to lymph nodes where the tumoral antigens are presented to T lymphocytes inducing their priming and activation. After that, activated T cells are intravasated in the bloodstream and penetrate the tumoral tissue where they recognize and destroy the tumoral cells, which release more tumoral antigens triggering another revolution of the cycle. During cancer progression, tumoral cells learn how to evade the immune system in practically every step of the cycle. From the development of a tumoral microenvironment, which compromises DC maturation due to different reasons such as a hypoxic environment, high lactic acid concentration, and high expression of cytokines (IL-10) or factors (VEGF, TGF-β), to the upregulation of certain membrane proteins as PD-L1 that hamper the killing capacity of T cells [30].

In this review, the use of nanocarriers designed to start over this virtuous cycle in cancer patients will be discussed, presenting some of the representative examples that illustrate the huge potential of nanomedicines to interact with immune cells and, therefore, to trigger potent self-sustained immune responses against tumors.

## 3. Nanoparticles to Enhance the Antitumoral Action of Innate Immune Cells

Tumoral cells present hundreds of mutations in coding regions of their DNA due to the acquisition of specific mutations in genes that control genome stability [31]. The mutator phenotype of cancerous cells provokes the apparition of altered proteins, tumor antigens, which can be recognized by immune cells as pathogen signals. There are two major types of immune cells: Innate immune cells as dendritic cells (DC), macrophages, or natural killers (NK), among others, and adaptive immune cells as B and T lymphocytes. Innate immune cells represent the first defensive line and are stationed in tissues checking the presence of pathogens or distress signals. In the case of malignant neoplastic tissue, NK can recognize tumoral antigens triggering the elimination of cancerous cells by the release of cytotoxic granulates and the secretion of specific cytokines, which induce the activation of adaptive immune responses [32]. Tumoral cells secrete immunosuppressive factors as TGF-β, which decreases the number of NK and their tumor-killing ability. Park et al. have developed a hybrid nanoplatform composed of a solid polymeric core, which contains interleukin-2 (IL-2) and TGF-β inhibitors, wrapped by a lipid bilayer to control the kinetic release of the payload [33]. Studies carried out with melanoma-bearing mice models showed that intratumoral administration of nanocarriers loaded with both drugs induced a potent tumor shrinkage increasing significatively the survival in comparison with the modest effect harvested in the case of nanocarriers loaded only with one of them, or the injection of free drugs. The authors found that the simultaneous and controlled release of these drugs increased the number of NK cells. The increase in the NK population was the main responsible of the antitumoral response because, when these nanocarriers were administered in NK-depleted mice, the therapeutic response was scarce. Instead, to transport IL-2, which is a sensible macromolecule, another interesting possibility is to deliver the gene, which encodes its synthesis. Thus, low molecular weight polyethyleneimine conjugated with cyclodextrins (CD) and folic acid, to enhance the nanoparticle cellular uptake, have been employed as gene delivery of IL-2 in melanoma cells [34]. Peritumoral injection of these nanosystems effectively suppressed tumoral growth in melanoma mice models by the activation of NK and T cells. NK presents tumortropic capacity being able to recognize and infiltrate into tumoral tissues. Additionally, once these cells arrive at malignant tissue, they can elicit an inflammatory response, which triggers the activation of other immune cells, enhancing their antitumoral response. The tumortropic capacity of NK can be transferred to nanoparticles employing their cell membrane to wrap the nanocarrier. Thus, poly-lactic-glycolic acid (PLGA) nanoparticles loaded with photosensitizers able to produce radical oxidative species under light irradiation were coated with the cell membranes of human NK [35]. Once the nanoparticles arrived at tumoral tissue, the irradiation with light at 660 nm induced immunogenic cell death (ICD) of tumoral cells. ICD is characterized by the release of damage-associated molecular patterns (DAMPs) as calreticulin (CRT), adenosine triphosphate (ATP), or high-mobility group protein B1 (HMGB1), which induces the activation of APC. The activation of these innate immune cells elicited a potent immunogenic response, which inhibited the growth of not only the primary tumor but also distant tumoral nodes by the abscopal effect. Nanoparticles coated with NK membranes have also been employed for the delivery of magnetic resonance contrast agents and near-infrared dyes for in vivo tumor imaging [36].

**Figure 2:**
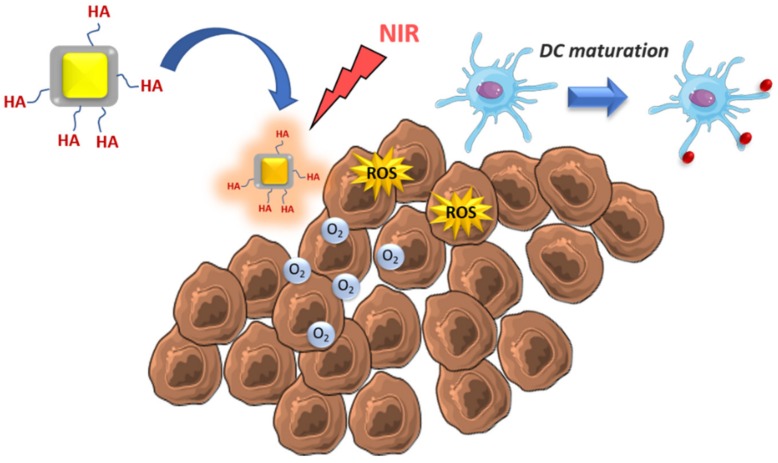
Mechanism of action of Au@MnO_2_ coated with hyaluronic acid (HA): Restoration of normoxic conditions and reactive oxygen species (ROS) generation under NIR trigger dendritic cells (DC) maturation.

DC are phagocytic cells that engulf cancerous cells and expose the captured tumoral antigens on their surface in order to present them to adaptive immune cells inducing the selection of specific antitumoral T lymphocytes. However, in many cases, simply the presence of tumoral antigens is not enough to induce a functional DC maturation, but it also requires the existence of danger or stress signals in the tumoral tissue [37]. In fact, when DC are exposed to tumor antigens but not to costimulatory signals, they can induce immunotolerance against the tumor, hampering the action of effector T cells. The administration of antigens and stimulating molecules to DC using nanoparticles has received huge attention in recent years in order to trigger sustained T cell-based immune responses [38,39]. In most cases, these nanocarriers are accumulated in lymph nodes by passive mechanisms or using targeting groups as mannose or ICAM ligands, among others [40,41,42]. Other alternatives consist of the use of nanoparticles, which induce ICD in the tumoral tissue. Zhao et al. have studied the antitumoral capacity of PLGA-PEG nanoparticles loaded with oxaliplatin (Ox) as ICD inducers, or gemcitabine (GEM) as non-ICD inducers, employing immunocompetent and immunodeficient mice models of pancreatic cancer [43]. Nanocarriers loaded with Ox induced strong immune responses with elevated excretion of DAMPs by tumoral cells, which caused enhanced DC activation and a higher proportion of infiltrated T lymphocytes. He and co-workers have recently developed core-shell nanoparticles for the treatment of advanced colorectal cancer by photodynamic therapy (PDT), which effect is also based on ICD induction of the cancerous cells [44]. These nanodevices are composed of a gold nanocage capable of generating ROS under near-infrared radiation (NIR), coated with a shell of manganese dioxide (Au@MnO_2_). This shell is in charge of the elimination of the hypoxic environment, usually present in this type of malignancy, which hampers the PDT efficacy. Thus, manganese shell generates oxygen within the tissue by the catalytic decomposition of H_2_O_2_ produced by the tumoral cells restoring normoxia, which enhances the ROS production within the tumor. ROS generation and normoxic oxygen condition favored DC maturation causing strong tumor growth inhibition (Figure 2). The capacity to recognize the tumoral cells of Au@MnO_2_ was achieved through the external decoration with hyaluronic acid (HA) due to the overexpression of this receptor (CD44) by colorectal cancerous cells.

The temperature increase is a danger signal, which facilitates DC maturation. This fact has been exploited using magnetic liposomes injected in the tumoral tissue through the generation of heat by alternative magnetic field exposition [45]. Active DC produces interleukin-12 (IL-12), which promotes T-cell response. Kim et al. have employed chitosan nanoparticles decorated with mannose on their surface for the transportation of IL-12 gen to DC [46]. Intratumoral injections of these nanoparticles in the colon adenocarcinoma murine model showed significant tumoral growth inhibition and angiogenesis elimination within the malignancy. The administration of immunostimulating agents in free form is usually associated with the apparition inflammatory toxicity. Therefore, their encapsulation within nanocarriers has been postulated to improve the safety of these therapeutic agents. Kwong et al. have attached on the surface of PEGylated liposomes anti-CD40, which bind to the co-stimulatory protein CD40 and certain oligonucleotide strands (CpG) that are recognized as pathogen-associated molecular patterns (PAMP) by Toll-like receptor (TLR) 9, both receptors located on the APC cell membrane [47]. Liposomes were injected directly in melanoma lesions of murine model inducing maturation of APC present both in the tumoral zone as well as in tumor-draining lymph nodes. The own nanocarrier by itself can induce APC maturation as is the case of cationic silica nanoparticles [48]. The uptake of these nanoparticles within tumoral cells induced cell necrosis mediated by the cationic surface of the nanocarriers, which provoked membrane rupture, and also by ROS generation. Necrotic tumoral cells release tumor-antigens prompting APC stimulation. The immunostimulatory effect was improved even more loading bis-(3′-5′)-cyclic dimeric guanosine monophosphate, a potent adjuvant that activates interferon production by the stimulator of interferon genes (STING) pathway. Another interesting alternative is to employ nanoparticles designed to capture tumoral antigens and deliver them specifically to APC in order to train them against the tumoral cells [49]. In this work, the authors have developed PLGA nanoparticles decorated with different groups: Amine polyethylene glycol and 1,2-dioleoyloxy-3-(trimethylammonium)propane, which can retain tumor-derived protein antigens (TDPAs) by electrostatic interactions and maleimide polyethylene glycol that capture TDPAs by the formation of covalent bonds between maleimide and the thiol group provided by cysteine residues of TDPA. The efficacy of this strategy was tested by employing mice models that carried bilateral melanoma flank tumors. One of the tumors was exposed to radiotherapy followed with nanoparticle injection, whereas the other one was shielded to avoid radiation exposition. Radiation-induced TDPA production, which was captured by the nanoparticles and delivered to APC. APC induced strong activation of CD8+ lymphocytes, which led to a significant antitumoral response in combination with anti-PD-1 therapy also in the non-treated tumor by the abscopal effect. Importantly, the animals were re-challenged with the tumor three months later, being able to reject it, which demonstrated the capacity of this strategy to induce durable memory effect against the malignancy. Another interesting approach to elicit a potent antitumoral immune response is to employ exosomes derived from APC [50]. Exosomes are nanometric vesicles that are excreted by living cells, which can compete in different roles such as cell-cell communications, transport large macromolecules as proteins, or RNA and immune response modulation. The exosomes gathered by mature DC co-cultured with tumoral cells have exhibited the capacity to stimulate cytotoxic T cell responses against the malignant cells [50].

Myeloid-derived suppressor cells (MSDCs) are immunosuppressive cells that are usually present in many solid tumors. Immature myeloid cells are generated in bone marrow being rapidly differentiated into several cell lines as granulocytes, macrophages, or DC. In certain pathological conditions, such as cancer, infection, or trauma, the differentiation is blocked, resulting in the formation of MSDCs. These cells play an important role in cancer development by suppression of T-cell function through different mechanisms such as the depletion of arginine by arginase I or ROS and peroxynitrite production, among others. Additionally, MSDC promotes regulatory T cells (T_reg_), which induce potent immunosuppression in cancerous tissues [51]. Due to these capacities, monotherapies designed to deplete MSDCs have received increasing attention in recent years. Liposomes loaded with gemcitabine were subcutaneously injected in melanoma-bearing mice, reducing the percentage of MSDC in the host [52]. This fact mitigated the immunosuppressive tumoral environment, which increased the efficacy of adoptive T-cell therapy. He et al. have reported that the administration of cationic polymers as cationic dextran and polyethyleneimine (PEI) promoted MSDC reprogramming of pro-tumoral phenotype M2 to anti-tumoral phenotype M1 restoring efficient immune responses in the tumoral tissue [53]. Zinc-doped iron oxide magnetic nanoparticles decorated with PEI were employed as a radioenhancer in glioblastoma therapy as a consequence of their good X-tray absorption capabilities [54]. The cationic coating facilitates the nanoparticle uptake in glioblastoma cells, causing their death by ROS production due to Fenton reaction within the lysosomes. Interestingly, the nanoparticles were also engulfed by MSDC cells present in the tumoral environment inducing M1 repolarization when the tumors were exposed to radiotherapy. The achievement of both effects, tumoral cell death, and MSDC repolarization, improved the survival rate in glioblastoma-bearing mice in comparison with animals exposed to nanoparticles or radiotherapy alone. The selective targeting of MSDC can be achieved by nanoparticle surface decoration with specific molecules as specific DNA aptamers [55]. An aptamer is a single strand DNA or RNA chain, which presents a 3D structure that can selectively bind to specific cell membrane receptors located on tumoral cells. In this example, Liu et al. attached a 74 base pair DNA aptamer on the surface of Dox-loaded liposomes, showing a significative enhancement in their uptake by breast cancer cells and also by MSDC. The depletion of these immune cells induced a major infiltration of cytotoxic T cells (CD8+) in the tumoral tissue. Wan et al. have recently developed pH-sensitive size-changeable micelles capable of delivering liver-X nuclear receptor (LXR) agonist RGX-104 and paclitaxel (PTX) to tumor stromal cells and tumor cells, respectively, which are two cell populations located in different areas of the tumor [56]. This system was composed of two types of micelles, which released their payloads under different pH conditions. One of them, released RGX-104 when the system arrived at the perivascular region of the tumoral tissue, where the pH was only slightly acidic (pH 6.8), whereas the other one was designed to release PTX once it was in the endosomes of the tumoral cell (pH 5.6), causing their destruction. RGX-104 was rapidly captured by MSDC, eliciting the ApoE pathway, which impaired their survival and, therefore, diminished the immunosuppressive tumoral environment enhancing the PTX effect. The capacity of MSDC to reach the tumoral tissue has been exploited using their membrane as nanoparticle coatings [57]. This biological coating provides stealth properties to the nanoparticle, making them invisible to immune cells and, at the same time, that provides tumor-homing capacities due to the presence of membrane receptors able to recognize the tumoral microenvironment. Nanoparticles can be employed not only to deplete or to reprogram MSDC but also to prevent their adhesion to premetastatic niche (PMN) one of the key steps in metastasis progression [58]. MSDC adhesion to PMN increases vascular permeability, immunosuppression, and facilitates circulating tumoral cells (CTCs) extravasation by different mechanisms, one of them being the overproduction of metalloproteinases as MMP-9. In this work, self-assembled micelles were decorated with P-selectin glycoprotein ligand-1 to bind to PMN. This fact facilitated the nanoparticle targeting of PMN at the same time that inhibited the recruitment of MSDC, which hampered PMN progression. The micelles were loaded with Dox and α- galactosylceramide (αGC) as immunopotentiator and their surface was additionally functionalized with phenylboronic acid to bind the sialic acid residues of tumoral cells, providing selectivity against the diseased cells. The hydrophobic core was composed of D-α-tocopheryl succinate (TOS), which inhibited the expression of MMP-9. The administration of these micelles prior to tumor surgery reduced the recruitment of MSDC in PMN, whereas the drug cocktail Dox/αGC/TOS enhanced specific antitumoral responses lowering postoperative metastasis recurrence prolonging the survival in tumor-bearing mice models.

**Figure 3:**
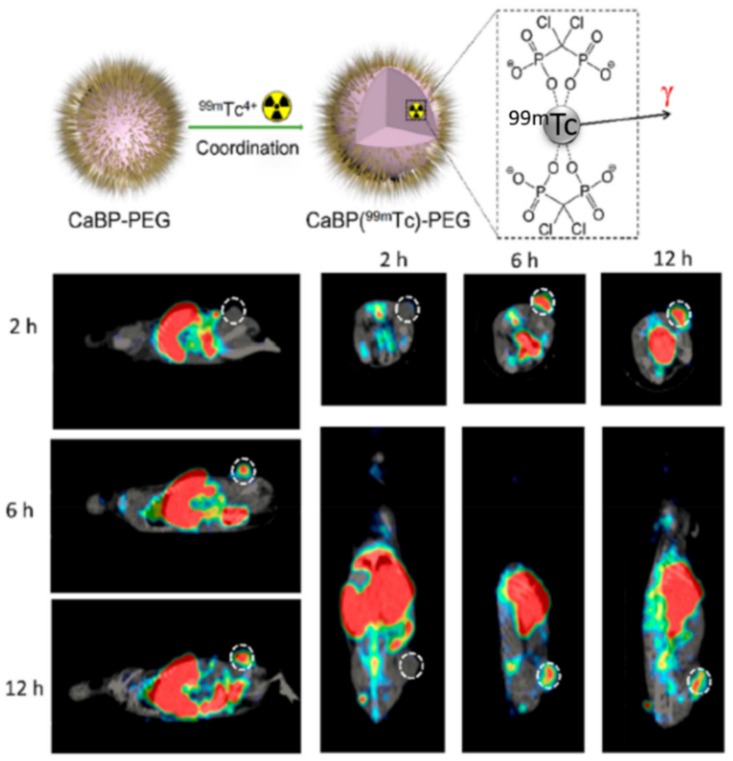
Single-photon-emission computed tomography (SPECT) images of CaBP(^99m^Tc)-PEG injected in mice bearing 4T1 breast tumors after 2, 6, and 12 h. White dotted circle corresponds to the tumoral area. This image is used with a slight modification from reference 67. Copyright 2018, American Chemical Society.

Finally, another type of innate immune cell, which plays a paramount role in tumor progression, is tumor associated macrophages (TAM). TAM intervenes at all stages of cancer development, from the beginning, where TAM contributes to tumor initiation, maintaining the inflammation of the tissue, to the consolidation and expansion of the tumor-inducing angiogenesis, immunosuppression, and supporting cell migration [59]. In many solid tumors, TAM can represent more than 50% of the tumor weight being a correlation between TAM density and poor patient prognosis. As in the case of MSDC, TAM can be widely classified into two groups: M1, which exerts antimicrobial and tumoricidal activities promoting inflammation and secreting effector molecules to activate adaptive immune cells and M2, which perform an anti-inflammatory and wound-healing role [60]. The tumoral microenvironment provokes the reprogramation of macrophages M1 to M2 phenotype, which supports tumor progression. Legumain-targeted liposomal nanoparticles loaded with hidrazinocurcumin were capable of inhibiting the signal transducers and activators of transcription 3 (STAT3) pathway leading to phenotype change from M2 to M1, restoring the antitumoral capacity of the macrophages [61]. Huang et al. have employed galactosylated cationic dextran to complex several oligonucleotide strands (CpG, anti-IL-10, and anti-IL-10RA) forming nanocomplexes, which were coated with PEG-histidine-modified alginate [62]. The PEG-histidine coating was removed when the nanoparticles reached the mild-acidic conditions of the tumor environment exposing the galactosylated surface. Then, the nanosystems were selectively engulfed by TAM due to the overexpression of sugar receptors on their surface. Once inside these cells, the nanoplatform released the oligonucleotide strands inducing IL-12 production and inhibiting IL-10, which restored the antitumoral capacity of these cells. A similar concept with pH-responsive PEG-sheedable has been employed to deliver PLGA nanoparticles to TAM in a selective manner [63]. In a recent paper, McParland et al. have studied the influence of macrophage phenotype in nanoparticle uptake [64]. The authors employed PEGylated gold nanoparticles as a nanosystem model found that the M2 phenotype was more active engulfing nanoparticles than M1. Furthermore, if the macrophages were polarized in the presence of pro-inflammatory stimuli as IFN-γ or lipopolysaccharides, the number of engulfed nanoparticles was reduced around 40% in comparison with cells polarized with regulatory stimuli as TGF-β/IL-10. Hypoxic regions of tumors usually recruit high amount of TAM, which are rapidly polarized to the M2 phenotype. Mannan-targeted manganese dioxide nanoparticles coated with hyaluronic acid (HA) were capable of inducing M2-M1 reprogramation [65]. The higher amount of M1-type macrophages enhanced the concentration of H_2_O_2_, which was catalytically decomposed to O_2_ by the manganese dioxide core restoring the normoxic condition of the tissue. Both effects, TAM reprogramation and normoxic condition, improved the effect of chemotherapy. ROS act as second messengers in M1 signal transduction of several pathways enhancing the antitumoral function of TAM. In a recent work, the photosensitizers indocyanine green (ICG) and titanium dioxide (TiO_2_) were encapsulated within mannose-targeted PLGA nanoparticles to generate ROS within TAM under NIR irradiation at 808 nm and UV light, respectively [66]. Ammonium bicarbonate was also co-encapsulated in order to induce the endosomal escape of the nanocarrier by the production of NH_3_ and CO_2_ under endosome acidification. ROS generation provokes M2-M1 macrophage repolarization which was more intense in the case of nanoparticles which contained ammonium bicarbonate that in the case of nanoparticles without this compound. This fact proved the better performance of the photosensitizers released in the cytosol in comparison with those retained within the endosomes. Interestingly, the total number of macrophages in the tumoral tissue was similar in the case of mice treated with nanoparticles and light than in the case of control mice treated with saline, which proved that this treatment skewed TAM from M2 to M1 instead of recruiting novel TAM. These M1 macrophages presented excellent T-cell-priming capacities provoking significant tumor shrinkage. In another interesting example, calcium bisphosphonate nanoparticles, which contained two radioisotopes: ^99m^Tc, which allowed to visualize the nanoparticle fate by single-photon-emission computed tomography (SPECT) imaging and ^32^P, as a therapeutic radioisotope, which destroyed tumoral cells by beta-emitting radiation, was reported as a theranostic nanoplatform (Figure 3) [67]. These nanoparticles (CaBP(^99m^Tc)-PEG) showed excellent tumor homing capacities, and once there, the dissolution of the calcium bisphosphonate core induced TAM depletion that in combination with the action of ^32^P radioisotope yielded a synergistic antitumoral effect without detectable toxicity. The most representative nanocarriers described in this section are listed in Table 1.

## 4. Nanoparticles to Enhance the Antitumoral Action of Adaptive Immune Cells

**Figure 4:**
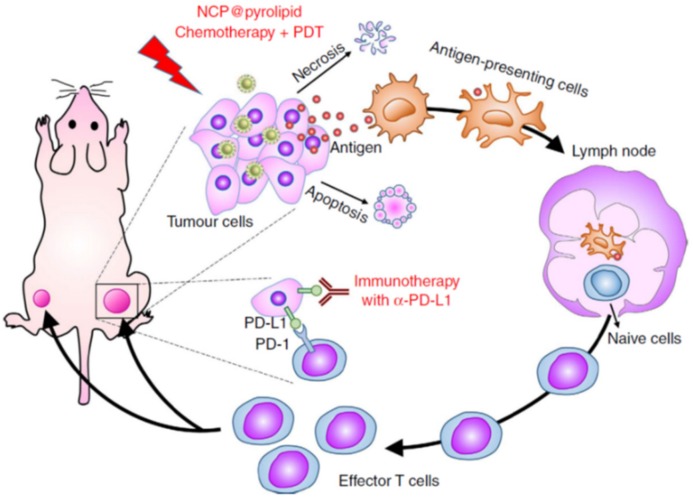
Combination therapy based on anti-PD-L1 and NCP@pyrolipid nanoparticles loaded with oxalilplatin and photosensitizers for triggering selective adaptive immune cell response in metastatic tumors. This image is used without modifications from reference 78. Copyright © 2020, Springer Nature.

When a pathological process is taken place within a tissue, sentinel immune cells trigger an inflammation process, which courses with the production of several agents as cytokines, chemokines, ROS, and metalloproteinases to promote the mobilization of more immune cells capable of combating the threat. DC engulf foreign agents exposing their antigens on the cell membrane and migrate to lymphoid organs to educate adaptive immune cells (B lymphocytes, CD4+ helper, T lymphocytes and CD8+ cytotoxic T lymphocytes (CTLs)) against the threat [68]. Mature DC present the captured pathogen antigens to these naive leucocytes amplifying those that carry the specific antigen-specific receptors by the clonal selection process. This step by step process presents a slower kinetic than innate response, but their correct development provides a robust and selective response against the threat. Importantly, during the clonal expansion, a subset of lymphocytes differentiates to long-lived memory cells that provide specific and rapid defense against the same threat during long periods of time [69]. In many tumors, the activation of innate immune cells happens more or less properly inducing the production of specific lymphocytes that travel to the tumoral tissue, but once arrived there, they find an immunosuppressive environment that compromises their function. The hallmarks of this immunosuppressive environment are varied such as: Loss of antigen presentation machinery mediated by tumoral cells that makes them invisible to T cells, presence of enzymes, which deplete metabolites which play a key role in T cell function (as IDO or arginase), presence of immunosuppressive mediators (as TGFβ or IL-10) or cells (T_reg_) and the expression on the tumor membrane of membrane receptors, which induce T cell apoptosis, is one of the most known of the programmed death ligand 1 (PD-L1) [70]. Peritumoral injection of nanoparticles loaded with potent immunostimulating agents as IL-2 has proved the efficacy to enhance the infiltration and activation of CD4+ and CD8+ T cells in melanoma models [34]. The combined delivery of IL-2 and TGF-β inhibitors induced a potent CD8+ T-cell infiltration, which delayed significatively the tumoral growth [33]. Huang et al. have reported the use of two types of nanoparticles to elicit antitumoral immune responses through the interaction with two cell populations, respectively [71]. In this work, liposome-protamine-hyaluronic acid nanoparticles were employed to deliver a siRNA to silence TGF-β expression in tumoral cells while lipid-calcium-phosphate (LCP) nanoparticles were engineered to stimulate DC through the release of tumor antigens and CpG as adjuvants. The administration of both nanosystems in melanoma-bearing mice inhibited the immunosuppressive environment yielding to higher infiltration of cytotoxic CD8+ T cells. Tumoral cells usually overproduce indoleamine-2,3-dioxygenase (IDO), an enzyme that degrades tryptophan to kynurenine. The lack of tryptophan in the tumoral tissue leads to T cell anergy. Wang et al. have synthesized layered double oxide nanoparticles (LDH) loaded with a potent IDO inhibitor (4-{[2-(4- bromophenyl)hydrazinyl]sulfonyl}benzoic acid) and the prodrug disuccinatocisplatin [72]. Once the nanocarriers were engulfed by tumoral cells, Pt(IV) of the prodrug was converted into highly toxic Pt(II) by the reductive cytosolic environment inducing tumoral cell apoptosis, whereas the released IDO inhibitor blocked the action of the enzyme that allowed the action of infiltrated cytotoxic T cells. A similar strategy has also been applied recently employing oxaliplatin and reduction-activatable IDO inhibitors (NLG919) that yielded significant CD8+ infiltration and reduction of immunosuppressive Treg population within the malignant tissue [73]. IDO inhibitors have been loaded in the pores of nanoparticulated hafnium (Hf)-based metal-organic frameworks (MOF) constructed with porphyrins, which also acted as photosensitizers [74]. Low-dose X-ray irradiation provoked the formation of ^•^OH radicals mediated by Hf clusters, which were rapidly transformed to ^1^O_2_ by the photosensitizers. The combination of ROS generation and IDO inhibitors released in the tumoral tissue induced a potent systemic antitumoral immune response, which reduced the tumoral growth not only in irradiated tumors but also in distant tumor nodes. Cheng et al. have synthesized an amphiphilic peptide that contained a peptide sequence cleavable by metalloproteinase-2 conjugated with a short D-peptide antagonist of PD-L1 [75]. These peptide-based nanoparticles formed by the self-assembly process were capable of loading IDO inhibitors (NLG919) inside them. The mild acidic conditions present in the tumoral environment provoked nanoparticle swelling, which rapidly suffered a collapse by the action of MMP-2 present there. This process released NLG919 that inhibited the IDO action and D-peptide antagonist of PD-L1, which enhanced the survival of cytotoxic T cells. T cells present in their surface a specific receptor called programmed cell death receptor PD-1, which binds to PD-L1 inducing T cell abnormalities and apoptosis [76]. The blockage of PD-L1 receptors on the tumoral cell surface has raised huge interest in the scientific community in order to unchain the antitumoral capacity of T cells, and, therefore, many different drugs have been developed to selectively inhibit this immunosuppressive pathway [77]. Unfortunately, PD-L1 blockage therapies alone have been not able to exert durable responses in many solid tumors with the exception of melanoma. He and co-workers have reported an interesting synergic effect, which appeared when tumor-bearing mice (metastatic colorectal cancer) were treated with anti-PD-L1 in combination with core-shell nanoparticles decorated with a photosensitizer on the shell (NCP@pyrolipid) and loaded with oxalilplatin within the core [78]. These nanoparticles reached the tumoral area by EPR effect, and once there, they were engulfed by the tumoral cells releasing oxalilplatin that triggered apoptosis with the exposition of calreticulin (CRT), a biomarker of ICD, which act as “eat me” signals for innate immune cells. Additionally, when the tumoral mass was exposed to light irradiation at 670 nm, ROS generated by the photosensitizers enhanced the presence of inflammatory cytokines TNF-α, INF-γ, and IL-6 in the tumoral mass at the same time that provoked higher tumoral cell elimination, both by apoptosis and by necrosis. This last cell death type improved the antigen-presentation capacity of innate immune cells. Thus, the administration of these nanoparticles caused an efficient activation and maturation of APC, which migrated to lymphoid organs to present the captured tumoral antigens to T and B cells. Finally, the administration of antibodies capable of blocking PD-L1 receptors on the surface of the tumoral cells allowed the selective destruction of the diseased cells not only in the primary tumoral mass but also in distant lesions, which were not exposed to light irradiation. This proved the suitability of this strategy to combat tumors in the metastatic stage (Figure 4).

**Figure 5:**
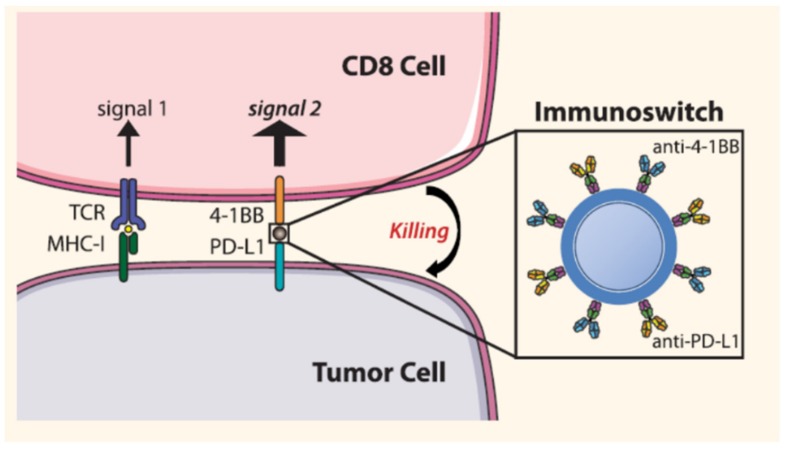
Immunoswitch based on double functionalized nanoparticles, which triggered T cell activation. This image is used without modifications from reference 84. Copyright © 2020, American Chemical Society.

Other types of nanoparticles that contained metallic atoms as copper [79] or zinc complexes [80] as photosensitizers have been employed to combine ICD induced by ROS generation triggered by PDT with PD-L1 inhibitors achieving promising outcomes in different solid tumors. ROS can be produced by employing drugs instead of PDT agents. Just to mention one example, dihydroartemisinin (DHA), a widely employed drug to treat malaria, contains a peroxide bridge that produces ROS in the presence of Fe^2+^ as a catalyst. This drug has been co-delivered in combination with oxalilplatin in a controlled manner in solid tumors employing coordination polymer-based nanoparticles [81]. DHA release induced ROS formation within the tumoral cells, which altered their cell surface composition, causing CRT exposition and DAMP secretion, which enhanced APC recruitment. The administration of these nanocarriers, in combination with anti-PD-L1, achieved long term antitumoral immunity against tumor re-challenge up to 3 months after the treatment. Another approach to block PD-1-PD-L1 recognition is to suppress PD-L1 expression by tumoral cells. Phung et al. have employed folate-targeted PLGA-PEI-PEG nanoparticles to deliver selectively to tumoral cells Dox and a specific microRNA (miR-200c) that inhibit PD-L1 expression [82]. Released Dox induced a strong ICD with enhanced expression and secretion of CRT and HMGB1 that are recognized as “eat me” and “find me” signals by APC, which produced a significant increase (around 22%) of mature and functional DC in tumor-draining lymph nodes. This effect enhanced the proliferation and infiltration of activated CD8^+^ T cells within the tumoral tissue. The release of miR-200c induced a potent inhibition of PD-L1 expression in the tumoral cells, which facilitated their destruction by the trained T cells. In another recent example, the PD-L1 silencing approach has been accomplished employing lipid-dendrimer-calcium-phosphate (TT-LDCP) nanoparticles able to transport siRNA that blocks the expression of the PD-L1 encoding gene and a plasmid that encodes the production of IL-2 [83]. This nanocarrier was engineered with a core composed of calcium-phosphate dendrimers, which strongly retained the oligonucleotide chains by electrostatic interactions. The nanocarrier surface was decorated with a specific peptide (SP94) to enhance the selectivity against hepatocellular carcinoma (HCC) cells. When these nanoparticles were engulfed by the tumoral cells, dendrimers and calcium phosphate core induced the endosomal escape of the nanocarrier by proton sponge effect and osmosis-mediated swelling process releasing the payload in the cytosol. PD-L1 silencing, in addition to induced IL-2 expression, improved CD8+ T cell infiltration in the tumoral tissue led to significant tumoral growth suppression in the murine orthotopic HCC model. Interestingly, this therapy was also capable of suppressing distal lung metastasis without inducing toxicity in the host. An interesting strategy to suppress PD-L1 at the same time that stimulates CD8+ T cell has been proposed by Schneck and co-workers employing iron oxide nanoparticles doubly functionalized with anti-PD-L1 and anti-4-1BB on the surface [84]. These nanoparticles act as a bridge between the tumoral cells and CD8+ cells through selective binding of the attached antibodies with their respective receptors located on both cells. Therefore, these nanoparticles blocked the immunosuppressive PD-L1 receptors placed on the tumoral cell while turned on the 4-1BB receptors placed on CD8+ surface which triggered a potent antitumoral activity against murine melanoma and colon cancer models (Figure 5). Importantly, these nanoparticles exerted the role of an immunoswitch that triggered CD8+ T cell activation without the need to involve cognate MHC-1 tumoral antigen recognition process mediated by TCR receptors. This fact enhanced the activation of polyclonal T cells with a wide repertoire of receptors that allowed to find the most efficient antitumoral response by the immune system. Regarding the use of antibody-decorated nanoparticles to activate T cells, different parameters such as surface density and spatial arrangement of these antibodies on the nanoparticle, as well as their amount, should be considered. A recent work has concluded that Janus-type nanoparticles with clustered ligands produced a better T cell activation than nanoparticles homogeneously decorated with the same amounts of ligands [85]. This result could be explained by the co-existence of multiple ligand-receptor interactions in the cluster conformation, which enhances the signal transduction in the lymphocyte. This work points out the necessity to study in detail the attachment process of the ligands on the particle surface in order to maximize the therapeutic efficacy.

Cytotoxic T-Lymphocyte Antigen 4 (CTLA-4) is a molecule expressed mainly on the surface of T lymphocytes, which exerts a regulatory function in immunity. An efficient antitumoral T cell response requires the presence of co-stimulatory signals; one of them is the interaction between CD28 receptors placed on the membrane of T cells with B7-1 and B7-2 receptors located on the surface of APC. CTLA-4 binds to B7 with higher intensity than CD28 downregulating immune response of T cells. Additionally, immunosuppressive T_reg_ usually overexpress CTLA-4 on their surface, hampering the stimulation of antitumoral T cells. Thus, antibodies as ipilimumab have been employed to block the CTLA-4-B7 binding process in order to remove this brake for the immune response against tumoral cells [86]. Furthermore, strong antitumoral responses have been achieved through intratumoral injection of anti-CTLA-4 and anti-PD-L1 in MB49 bladder murine tumors models proving the antitumoral capacity of strategies focused on the simultaneous blockage of both immune checkpoints [87]. Chen and co-workers have combined anti-CTLA-4 administration with photodynamic therapy employing PLGA nanoparticles loaded with indocyanine green (ICG) as a photosensitizer and the Toll-like-receptor-7 agonist, imiquimod (R837), as an adjuvant [88]. These nanoparticles were intratumorally injected in mice-bearing subcutaneous breast 4T1 tumors, and then, the lesions were irradiated with NIR at 808 nm reaching local temperatures up to 60 °C. After 3 days of tumor’s thermal ablation, the lymph nodes were collected and analyzed by flow cytometry showing an elevated level of DC maturation (72%) higher than in the case of non-irradiated tumors (around 60%). Additionally, higher secretions of immunostimulating cytokines as TNF-α, IL-12p70, and IL-6 was found in irradiated mice. In a further experiment, mice were inoculated with breast cancer (4T1) and colorectal cancer (CT26) cells, respectively, in both flanks with 1 week of difference between each inoculation. At day 8, the primary tumors were removed by surgery or by thermal ablation with the PLGA-ICG-R837 nanoparticles plus NIR radiation. Then, 3 doses of anti-CTLA-4 per mouse were injected in the tail every 3 days after thermal ablation. The stronger tumoral growth inhibition of the secondary tumors was observed in the animals in which the tumors were removed by thermal ablation followed by anti-CTLA-4 treatment. Interestingly, the tumors that were removed by surgery showed a worse response even when anti-CTLA-4 was administered, which proved the virtuous synergism between the immunostimulating capacity of PLGA-ICG-R837+NIR and anti-CTLA-4 therapy. The same research group has reported a similar system that utilized upconversion nanoparticles that contained chlorine6 (Ce6) as a photosensitizer in order to generate ROS under longer NIR wavelengths, 980 nm instead of 808 nm [89]. The main advantage of this type of radiation is its higher penetration capacity in living tissues that allows the application of this strategy to deeper malignancies.

One of the residents of tumoral tissues that play a leading role supporting the immunosuppressive tumoral environment are T_regs_. These cells are a specialized subpopulation of T cells that exert immunosuppressive functions favoring the tolerance to self- and nonself-antigens in order to maintain the homeostasis of tissues. Their action is caused by different mechanisms as inhibition of stimulatory CD80 signals in DC by overexpression of CTLA-4, secretion of cytokines (IL-10, TGF-β), consumption of IL-2, metabolic alterations of tryptophan or arginine or direct depletion of cytotoxic T cells [90]. Several studies point out the association between poor prognosis and higher Treg infiltration in the tumor [91]. Therefore, the design of strategies targeted to deplete these cells employing antibodies [92] or drugs as Imatinib (IMT) [93] has led to significant enhancement in antitumoral immune responses, although they are not free of drawbacks as side toxicity or solubility problems. Ou et al. have developed core-shell PLGA@lipid nanoparticles capable of delivering IMT in a selective and safe manner to Treg thanks to the surface decoration with a specific peptide tLyp1 [94]. This peptide presents high affinity by Neuropilin-1 (Nrp1) receptor, which is widely expressed on Treg but scarcely present in T effector cells. The combined treatment with these IMT-loaded nanocarriers and anti-CTLA-4 induced a selective T_reg_ depletion in melanoma-bearing mice, which reverted the immunosuppressive environment leading to a higher CD8+ T cells accumulation and activation in the tumoral tissue. Chen and co-workers have employed small polymer-coated iron oxide nanoparticles (around 30 nm of diameter) to destroy Treg in breast tumor murine models by photothermal therapy (PTT) [95]. It is worthy to point out here the difference between PDT, which employs photosensitizers to produce ROS in the presence of certain light wavelengths and PTT, that provoke a temperature increase in the tissue without generating ROS. In the first approach, its efficacy is highly correlated with the presence of oxygen that limits its efficacy in hypoxic tissues, as is usually the case of tumor cores. The effect of PTT is independent of oxygen concentration and is only based on the cellular damage caused by the temperature increase. Therefore, it is a useful alternative for the treatment of hypoxic tumors. In this work, the iron oxide nanoparticles accumulated in the tumoral tissue provoked that temperature locally raised up to more than 50 °C under NIR exposition at 885 nm during 10 min. After one irradiation round, which released tumor antigens due to the destruction of malignant cells in the tissue, the T_reg_ population became dominant because these cells respond more efficiently to antigens exposition than CD8+ T cells. A second irradiation round 24 h later destroyed the tumor-homing replenished T_regs_ whereas barely affected to freshly activated cytotoxic CD8+ T cells, which did not have time enough to reach the tumoral tissue. PTT mediated with these iron-oxide nanoparticles in combination with anti-CTLA-4 induced a significant inhibition in the tumor growth and, even more important, provided a memory effect that protected the mice against tumor re-challenge.

Tumors secrete different factors as collagen or thrombin, which promote platelet activation. Platelets exert a key role in tumor development and progression promoting metastasis, angiogenesis, chemoresistance, and the release of soluble factors such as sphingosin-1-phosphate or serotonin that maintain a strong tumor vessel barrier which limits the infiltration of T cells in the tumoral tissue, among others [96]. An effective adaptive immune response requires the proper trafficking of T cells to the tumoral tissue. Zhou and co-workers have reported that perfluorotributylamine (PFTBA)-albumin nanoparticles (PFTBA@Alb) showed high platelet inhibitory capacity that increased the permeability of tumoral blood vessels to CD8+ T cell infiltration enhancing the effect of anti-PD-L1 therapy [97]. Finally, the tumor-homing capacity of platelets has been employed to deliver iron-oxide nanoparticles coated with platelet membrane in order to destroy tumoral lesions by local PTT taking advantage of the selective accumulation of the nanoparticles in the tumor [98]. Moreover, their biodistribution was monitored in real time by magnetic resonance imaging due to the contrast enhancer properties of iron oxide nanoparticles. The most representative nanocarriers described in this section are represented in Table 2.

## 5. Conclusions

The development of smart nanoparticles capable of transporting therapeutic agents that exhibit very different natures from small drugs, as is the case of many chemotherapeutic compounds, to large macromolecules as proteins and oligonucleotide chains have provided an incredibly powerful tool to oncologists. During the last decades, thanks to the creativity and hard work of the scientific community, a myriad of different nanocarriers endowed with remarkable properties have been reported. Thus, these nanocarriers can be engineered with the capacity to release their cargo on-demand (stimuli-responsive behavior), ability to recognize their target cell (cellular targeting), and even certain organelles inside them (organelle targeting) and can be monitored in real-time during their journey through the patient (imaging). Despite these huge efforts, the clinical effect of nanomedicines has not been as satisfactory as previously thought with only a scarce bunch of nanomedicines in the market. The existence of important barriers that reduce the number of nanoparticles that reach the cancerous tissue and that hamper their homogeneous distribution inside there seriously compromises the efficacy of nanoparticle-based therapies focused on destroying tumoral cells. In the last years, the need to switch the nanoparticle’s target to tumoral cells to the cells of the immune system is gaining huge attention in the nanomedicine community. After years struggling against the fine-tuned capacity of immune cells to capture nanoparticles, the current paradigm is changing to take advantage of this fact through the development of nanocarriers designed to interact with immune cells in order to mobilize them against the tumor. Despite the promising results harvested with this novel approach, prudence is required not to create another nanomedicine hype [99]. The immune system is really complex machinery, and more knowledge is required to understand the implications of manipulating one specific cell population or another. The use of nanoparticles to manipulate and control the subtle interactions between immune cells with themselves and between immune and tumoral or stroma cells, poses a huge challenge where the scientific community should work in close contact between different disciplines as immunology, oncology, biology, chemistry, and engineering, just to quote a few of them. There is no doubt about the capacity of immune cells to recognize nanoparticles. This fact can be employed to orchestrate an efficient antitumoral response, which eliminates the tumoral cells in the whole organism making the nanoparticles excellent allies in the fight against cancer.

## Figures and Tables

**Table 1:** Selected examples of nanoparticles developed to act in innate immune cells.

Mechanism of Action – Immune Cell Target	Nanoparticle Type	Payload	Tumor Model	Ref
Enhance NK population	Liposomal-polymer core-shell	IL-2 and TGF-β inhibitors	Melanoma	[33]
Recruitment and activation NK and T cells	Polyethylenimine-β-cyclodextrin	IL-2 gene	Melanoma	[34]
Production of DAMPs – NK and APC activation	PLGA	NIR photosensitizers	Breast cancer	[35]
ICD of tumoral cells – DC activation and high T cell infiltration	PLGA-PEG	oxaliplatin	Pancreatic cancer	[43]
ROS generation by NIR exposition – DC activation	Core-shell Au@MnO_2_	-	Colorectal cancer	[44]
DC activation	Chitosan	IL-12	Colon adeno-carcinoma	[46]
DC activation	PEGylated -liposomes	anti-CD40 and CpG	Melanoma	[47]
Capture of tumor antigens after radiotherapy – DC activation	PLGA	Amino- and maleimide groups	Melanoma	[49]
MSDC depletion	Liposomes	gemcitabine	Melanoma	[52]
Tumoral cell elimination by ROS and MSDC repolarization	Zinc-doped iron oxide-PEI	-	Glioblastoma	[54]
MSDC depletion	Aptamer-liposomes	Dox	Breast cancer	[55]
Tumoral cell elimination and MSDC depletion	pH-sensitive micelles	RGX-104 and PTX	Breast cancer	[56]
Reduction of MSDC recruitment after tumor surgery	self-assembled micelles	Dox, αGC and TOS	Lung metastasis	[58]
TAM repolarization M2-M1 by SAT3 inhibition	Liposomes	Hidrazinocurcumin	Breast cancer	[61]
TAM repolarization M2-M1	Dextran PEG-histidine-modified alginate	CpG, anti-IL-10 and anti-IL-10RA	Hepatoma	[62]
TAM repolarization M2-M1	Core-shell manganese dioxide@HA	Dox (coadministration)	Breast cancer	[65]
TAM repolarization M2-M1 by ROS generation under NIR	PLGA	ICG, TiO_2_ and NH_4_HCO_3_	Breast cancer	[66]
TAM depletion by calcium bisphosphonate dissolution and tumor imaging by SPECT	calcium bisphosphonate	^99m^Tc and ^32^P radioisotopes	Breast cancer	[67]

**Table 2:** Selected examples of nanoparticles developed to act in adaptive immune cells.

Mechanism of Action – Immune Cell Target	Nanoparticle Type	Payload	Tumor Model	Ref
Elimination of immunosuppressive environment - higher infiltration CD8+ T cells	liposome-protamine-hyaluronic acid and lipid-calcium-phosphate	siRNA to silence TGF-β and tumor antigens/CpG	Melanoma	[71]
Tumoral cell elimination and IDO inhibition - Higher infiltration of T cells	Layered double hydroxide Mg/Al	IDO inhibitors and disuccinatocisplatin	Cervical cancer	[72]
IDO inhibition and ROS generation under X-rays - T cell activation, abscopal effect and tumor rechallenge resistance	Hafnium (Hf)-based MOF	IDO inhibitors and porphyrins	Several tumor models	[74]
Enhanced survival of T cells	Self-assembled amphiphilic peptide	IDO inhibitors and PD-L1 antagonist	Melanoma	[75]
Higher expression of CRT, TNF-α, INF-γ and IL-6 - higher APC maturation and higher T cell activation and infiltration	Core-shell coordination polymers	Photosensitizer/oxalilplatin and anti-PD-L1	Colorectal cancer	[78]
ROS generation which enhances expression CRT - T cell activation, memory effect	coordination polymer	Dihydroartemisinin and anti-PD-L1	Colorectal cancer	[81]
ICD tumoral cells, higher T cell activation and infiltration	PLGA-PEI-PEG	Dox and microRNA that silence PD-L1	Colon adenocarcinoma	[82]
Improve CD8+ T cell infiltration	Lipid-dendrimer-calcium-phosphate	siRNA that silence PD-L1 and plasmid encoding IL-2	Lung metastasis	[83]
Blocks PD-L1 and stimulate T cell with anti-4-1BB	Iron oxide	Anti-PD-L1 and anti-4-1BB	Melanoma	[84]
Thermal ablation under NIR -DC stimulation; T cell activation; abscopal effect	PLGA	Photosensitizer/imiquimod and anti-CTLA-4	Breast and colon cancer	[88]
Selective depletion T_reg_ by targeting neuropilin-1 receptor with tLyp1	PLGA@lipid decorated with tLyp1	Imatinib combined with anti-CTLA-4	Melanoma	[94]
PTT by NIR to destroy T_reg_	Polymer-coated iron oxide	Combined with anti-CTLA-4	Breast cancer	[95]
Platelet depletion; increase blood vessel permeability of T cells	perfluorotributylamine (PFTBA)-albumin	Combined with anti-PD-L1	Colorectal and melanoma	[97]
